# Improving outcomes of acute kidney injury using mouse renal progenitor cells alone or in combination with erythropoietin or suramin

**DOI:** 10.1186/scrt225

**Published:** 2013-06-18

**Authors:** Xiao Han, Li Zhao, Guodong Lu, Junke Ge, Yalin Zhao, Shulu Zu, Mingzhen Yuan, Yuqiang Liu, Feng Kong, Zhiying Xiao, Shengtian Zhao

**Affiliations:** 1 Department of Urology, The Second Hospital, Shandong University, Jinan, PR China; 2 Department of Microbiology/Key Laboratory for Experimental Teratology of Chinese Ministry of Education, School of Medicine, Shandong University, Jinan, PR China; 3 University of Oregon, Eugene, USA

**Keywords:** Adult kidney stem cell, Cell therapy, Erythropoietin, Suramin

## Abstract

**Introduction:**

So far, no effective therapy is available for acute kidney injury (AKI), a common and serious complication with high morbidity and mortality. Interest has recently been focused on the potential therapeutic effect of mouse adult renal progenitor cells (MRPC), erythropoietin (EPO) and suramin in the recovery of ischemia-induced AKI. The aim of the present study is to compare MRPC with MRPC/EPO or MRPC/suramin concomitantly in the treatment of a mouse model of ischemia/reperfusion (I/R) AKI.

**Methods:**

MRPC were isolated from adult C57BL/6-gfp mice. Male C57BL/6 mice (eight-weeks old, n = 72) were used for the I/R AKI model. Serum creatinine (Cr), blood urea nitrogen (BUN) and renal histology were detected in MRPC-, MRPC/EPO-, MRPC/suramin- and PBS-treated I/R AKI mice. E-cadherin, CD34 and GFP protein expression was assessed by immunohistochemical assay.

**Results:**

MRPC exhibited characteristics consistent with renal stem cells. The features of MRPC were manifested by Pax-2, Oct-4, vimentin, α-smooth muscle actin positive, and E-cadherin negative, distinguished from mesenchymal stem cells (MSC) by expression of CD34 and Sca-1. The plasticity of MRPC was shown by the ability to differentiate into osteoblasts and lipocytes *in vitro*. Injection of MRPC, especially MRPC/EPO and MRPC/suramin in I/R AKI mice attenuated renal damage with a decrease of the necrotic injury, peak plasma Cr and BUN. Furthermore, seven days after the injury, MRPC/EPO or MRPC/suramin formed more CD34^+^ and E-cadherin^+^ cells than MRPC alone.

**Conclusions:**

These results suggest that MRPC, in particular MRPC/EPO or MRPC/suramin, promote renal repair after injury and may be a promising therapeutic strategy.

## Introduction

Acute kidney injury (AKI) mainly develops following ischemic or toxic insults and is characterized by acute tubular injury and renal dysfunction [1,2]. Modern dialysis techniques, such as intermittent or continuous renal replacement therapy, are used in the treatment of AKI, but the syndrome is still characterized by a high mortality and morbidity rate [3]. Thus, it is urgent for us to identify new drugs and find novel therapeutic strategies.

Recently, stem cell therapy has been proposed as a promising alternative in the treatment of AKI [4-8], due to the highly versatile response of cells to their environment. The potential use of stem cells in regenerative medicine to treat kidney diseases represents a critical clinical goal [9]. Mounting evidence indicates that stem cells from different sources have therapeutic potential for AKI, including bone marrow-derived stem cells [4-7], embryonic stem cells, induced pluripotent stem cells, human amniotic fluid stem cells [10], human cord-blood stem cells [11] and resident renal stem cells [8]. Among these stem cells, little is known about renal stem cells in the treatment of AKI, because their localization, markers, function and mechanism are still not fully understood. Recent study focuses on an important role of renal stem cells in the treatment of AKI by the mechanism of differentiating into renal tubule cells [12-14]. Especially, mouse renal stem cells accelerate renal regeneration and prolong survival after AKI by differentiating into renal tubule cells and vessel endothelial cells with the expression of E-cadherin and CD34 [15]. This potentially gives a clue to the development of regenerative medicine in the treatment of human renal diseases. Although many efforts have been made to investigate renal stem cells in the treatment of AKI, therapy with renal stem cells for AKI treatment needs more research.

Besides stem cell-based therapy, drug therapy is also applied in the recovery of renal ischemia/reperfusion (I/R) injury. Thus, exploring new drugs or novel pharmacological effects of known drugs in the treatment of AKI is urgent. Recently, erythropoietin (EPO) and suramin were intensely studied in the treatment of AKI for their novel pharmacological effect. EPO may have tissue-protective properties in addition to its well-known erythropoietic function [16]. Song YR *et al*. [17] report that preventive administration of EPO could prevent AKI and improve postoperative renal function. EPO may preserve kidney integrity and reinforce the regeneration of tubular epithelium by anti-apoptotic and anti-inflammatory features [18]. Suramin, a polysulfonated naphthylurea usually given in humans in the treatment of trypanosomiasis, is reported to accelerate recovery from renal dysfunction caused by IR injury in mice [19,20]. The mechanisms remain incompletely understood and may be related to prevention of apoptosis, inhibition of inflammatory cytokine generation and facilitating epithelial cell hyperplasia [19].

In this study, we explored the different effects of mouse adult renal progenital cells (MRPC) alone or MRPC/EPO or MRPC/suramin in the treatment of AKI. Mouse renal MRPC which were isolated from adult GFP mice, possessed features consistent with renal stem cells. Injection of these MRPC, MRPC/EPO, or MRPC/suramin could rescue renal damage in I/R AKI C57BL/6 mice, followed by formation of CD34^+^ and E-cadherin^+^ cells. More pronounced protection of renal function was found in mice treated with MRPC/EPO or MRPC/suramin. Thus, MRPC, particularly MRPC/EPO or MRPC/suramin, might be a promising therapeutic target for the treatment of AKI.

## Methods

Experiments were carried out on 72 male C57BL/6 mice, with weights ranging from 27 to 32 g at the time of ischemia. C57BL/6-gfp mice were bought from the experimental animal center of the Fourth Military Medical University. All animal procedures were approved by the animal ethics committee of Shandong University (Jinan, China) and followed the Guide for the Care and Use of Laboratory Animals published by the U.S. National Institutes of Health (NIH Publication No. 85–23, revised 1996).

### Cell isolation and culture

MRPC were isolated from the renal cortex of eight-week-old C57BL/6-gfp transgenic mice (Fourth Military Medical University, Xian, China) using a previously reported approach [8,21]. Briefly, the kidney was perfused *in vivo* with PBS to wash out blood and was then dissected. The renal capsule and medulla tissue were removed and digested with 0.125% type_IV collagenase (Sigma-Aldrich, St Louis, MO, USA) and 0.25% trypsin at 37°C for 30 minutes with gentle shaking. After resuspension in (D)MEM/F12 Sigma-Aldrich), the fraction was filtered through a 200 μm mesh (BD Biosciences, San Jose, CA, USA) to remove undigested tissue, and then a 40 μm mesh was used to remove smaller renal tubules and cell aggregates. The filtered fraction was washed with (D)MEM/F12 containing 10% fetal bovine serum (FBS) (Sigma-Aldrich). To exclude autofluorescence of isolated cells, the level of autofluorescence was detected in similar cell preparations from C57BL/6 mice under a fluorescence microscope. To avoid cell–cell contact, GFP-positive cells were plated at low density (300 cells/cm^2^) on fibronectin coated culture flasks in the (D)MEM/F12 culture medium containing 10% FBS, 100 U/ml of penicillin, 100 μg/ml of streptomycin, and incubated at 37°C in the presence of 5% CO_2_.

### Characterization of MRPC

#### Immunocytochemistry of MRPC

Cells growing on a poly-l-lysine coated 24-well plate were washed three times with PBS and fixed in 4% paraformaldehyde for 30 minutes. Cells were permeabilized with 0.1% Triton X-100 PBS for 20 minutes and then blocked with 4% goat serum for one hour. Then cells were incubated with primary antibodies for one hour at room temperature in the absence of sunlight. The following primary antibodies were used: mouse monoclonal anti-Oct-4 (Chemicon, Billerica, MA, USA, MAB4419, 1:200), rabbit polyclonal anti-Pax2 (Covance, Emeryville, CA, USA, PRB-276P, 1:400), rat monoclonal anti-E-cadherin (Chemicon, MABT26, 1:200), mouse polyclonal anti-vimentin (Chemicon, MAB3400, 1:200) and mouse monoclonal anti-alpha smooth muscle actin (α-SMA) antibody (Sigma-Aldrich, A2547, 1:600). After three washes with tris-buffered saline (TBS), cells were incubated with alexa 594-conjugated secondary antibodies (Zhongshan Goldenbridge, China) in PBS. 4,6-Diamino-2-phenyl indole (DAPI) was used for nuclear counterstaining. After washing, slides were mounted with a cover slip in Glycergel Antifade Medium (Dako, Carpinteria, CA, USA). Negative controls were performed using non-immune IgG instead of the primary antibodies. Images were obtained using an Olympus fluorescence microscope. Two independent investigators evaluated the number of Oct-4-, Pax-2-, vimentin- and α-SMA-positive MRPC by counting three randomly selected high-power fields.

#### Differentiation in vitro

A total of 10^5^ intact cells were plated onto a six-well plate for differentiation of cloned MRPC *in vitro*. Adipocyte differentiation was induced in (D)MEM/F12 culture medium containing 1-methyl-3-isobutylxanthine, 10^-6^ M dexamethasone, 10 μg/ml insulin, and stained with saturated Oil-Red O (Sigma-Aldrich) in 60% isopropanol to detect oil droplets two weeks later [22]. Osteogenic differentiation was induced in (D)MEM/F12 culture medium containing 50 μg/ml ascorbic acid, 10 mM β-glycerophosphate and 100 nM dexamethasone. Alizarin red staining was used to detect calcium deposition three weeks later [22].

### Reverse transcription PCR

Total RNA was extracted from MRPC or mesenchymal stem cells (MSC) using Trizol Reagent (Invitrogen, Carlsbad, CA, USA) and 2 μg of total RNA was reverse transcribed into cDNA with oligo-dT primer and reverse transcriptase (Fermentas, Vilnius, Lithuania). PCR was performed with specific primer sets at 95°C for 5 minutes, 95°C for 30 seconds, 60°C for 30 seconds, and 72°C for 30 seconds (30 cycles) followed by 72°C for 10 minutes. The forward and reverse primers were designed by Primer 5 for mouse gene: TCC CAG TGT CTC ATC CAT CA (forward primer) and GTT AGA GGC GCT GGA AAC AG (backward primer) for Pax-2, CAC GAG TGG AAA GCA ACT CA (forward primer) and AGA TGG TGG TCT GGC TGA AC (backward primer) for Oct-4, ACC ACA GAC TTC CCC AAC TG (forward primer) and CGG ATT CCA GAG CAT TTG AT (backward primer) for CD34, CCA CCA GGG ACT GAC AAG TT (forward primer) and TGT AAT TTG TTT GGG CAC GA (backward primer) for CD45, CCA TCA ATT ACC TGC CCC TA (forward primer) and TTC CTG GCA ACA GGA AGT CT (backward primer) for Sca-1, ATT TTC TG GGC AGG AAG TT (forward primer) and ACG TCA GAA CAA CCG AAT CC (backward primer) for CD106, CGC TCT CCT GCT CTC AGT CT (forward primer) and GCA CGT GCT TCC TCT TCT CT (backward primer) for CD90, AGA ACT GGA GAA GTG TGG CTG TGA CC (forward primer) and TGT ATG TGG CTT GAA CTG TGC ATT CCG (backward primer) for Wnt-4, ACA TCC GAC TTC CAA GAC AGC ACA C (forward primer) and TTG CAG CCA GAC CTC TGA AAT TCT G (backward primer) for WT-1, and ACG GCA CAG TCA AGG CTG AG (forward primer) and GGA GGC CAT GTA GAC CAT GAG G (backward primer) for glyceraldehyde-3-phosphate dehydrogenase. PCR products were subjected to 2% agarose gel electrophoresis, stained with ethidium bromide, and visualized under UV transilluminator.

### Effect of MRPC on renal protection after acute ischemic injury

#### Study design

Twenty-four mice were randomly divided into controls (positive control) or either of the three treatment arms (MRPC, MRPC/EPO or MRPC/suramin). Animals were housed at a constant temperature and humidity, with a 12:12-hour light–dark cycle. At days 0 (pre-ischemia), 1, 2 and 3, blood samples were collected for the measurement of serum creatinine (Cr) and blood urea nitrogen (BUN). Cr and BUN concentrations were detected by the Jaffe method (Beckman Coulter Synchron LX System; Beckman Coulter Inc., Brea, Calif., USA). Then, the mice were sacrificed at day 7. An additional 48 mice were used to observe the early changes in the kidney after injury; 24 mice (n = 6 in each group) were sacrificed at day 2, and the other 24 mice (n = 6 in each group) were sacrificed at day 4. Bilateral kidneys were obtained and fixed with formalin followed by paraffin embedding. Sections were stained with H & E and studied histologically for morphologic changes induced by ischemic injury. A grading scale (range: 0 to 4) for assessment of acute tubular necrosis developed by Jablonski *et al*. was used for the histopathological assessment of acute ischemic injury [23]. In addition, immunohistochemistry assays were performed with anti-GFP antibodies to detect and localize the infused stem cells in the tissue as well as the expression level of E-cadherin and CD34 after treatment.

#### Surgical procedure

Mice were anesthetized with an intraperitoneal injection of phenobarbital (150 μg/g). An abdominal midline incision was made to expose the kidneys and nontraumatic vascular clamps were used to clamp both renal pedicles for 30 minutes at room temperature. After visual reflow of both kidneys, 50 μl of cell suspensions containing 5 × 10^5^ MRPC in PBS or MRPC/EPO (administration of 5 × 10^5^ MRPC in 50 μl PBS and 1 μg/kg of EPO) or MSC/suramin (administration of 5 × 10^5^ MRPC in 50 μl PBS and 1 mg/kg of suramin) were injected immediately and slowly through the tail vein after surgery. Mice in the control group received 50 μl of PBS only.

#### Immunohistochemistry

Fixed mouse kidney consecutive sections were deparaffinized in xylene and rehydrated through a graded ethanol series to water. After blocking with 4% normal goat serum in PBS, the slides were incubated with primary antibodies overnight at 4°C, biotinylated secondary antibody for 20 minutes. The following primary antibodies were used: rat monoclonal anti-E-cadherin (Chemicon, MABT26, 1:200), rat monoclonal anti-CD34 (Abcam, Cambridge, MA, USA, AB8158, 1:500) and mouse monoclonal anti-GFP (Chemicon, MAB3580, 1:1000).

### Statistical analysis

Data are shown as means ± SD. Comparison between groups was evaluated by two-way analysis of variance (ANOVA) or unpaired t test. *P* <0.05 was considered statistically significant.

## Results

### Isolation and culture of fluorescent MRPC

**Figure 1:**
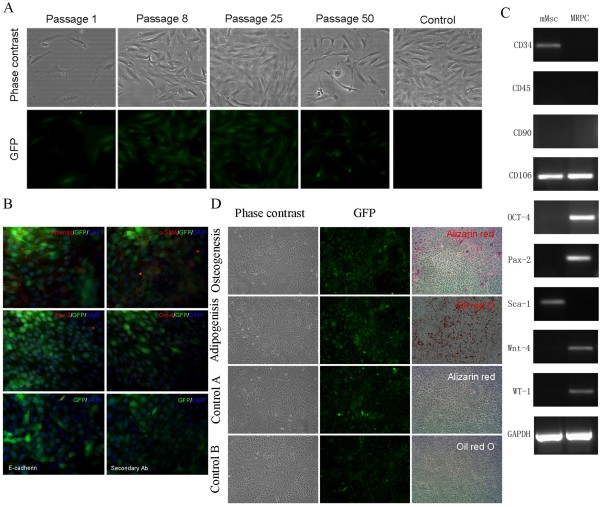
**Characteristics and differentiation potency of MRPC.** (**A**) Morphology of MRPC isolated from adult mouse kidney. Morphology of the cells after 1 passage (4 days), 8 passages, 25 passages, and 50 passages is shown by phase-contrast microscopy and immunofluorescence microscopy. After eight passages, the cells are monomorphic with a spindle-shape morphology, containing large nucleus and fluorescence. MRPC cultured to confluence at passage 25 do not overlay and maintain fluorescence. Autofluorescence was negligible in the control group. (magnification 200×) (**B**) Immunofluorescence microscopy of fluorescent MRPC (green) stained with the following antibodies (red): anti-Oct-4 antibodies, anti-Pax-2 antibodies, an anti-alpha smooth muscle actin antibody (α-SMA), an anti-vimentin antibody, an anti-E-cadherin antibody and secondary antibody only (magnification 400×). (**C**) Gene expression of mMSC (bone marrow) and MRPC detected by RT-PCR. (**D**) Mutilineage differentiation of MRPC. Phase-contrast microscopy and immunofluorescence microscopy of MRPC that were incubated under culture conditions that induced differentiation into osteoblasts and adipocytes. Control A was osteogenic differentiation and control B was adipogenic differentiation (magnification 100×). MRPC, mouse renal progenitor cells; MSC, mesenchymal stem cells, α-SMA.

MRPC were isolated from six- to eight-week old C57BL/6-gfp mice. Cells from six- to eight- week old C57BL/6 mice were used as controls for autofluorescence detection. Autofluorescence was negligible in cells from C57BL/6 mice as detected by fluorescence microscopy (Figure 1A). Dispersed cells from C57BL/6-gfp mice became monomorphic and had a spindle-shaped appearance after four weeks of culture (Figure 1A). These cells containing a large nucleus and scant cytoplasm, expressed green fluorescence at different passages (Figure 1A). After more than 50 passages, there was no evidence of senescence in some clones. MRPC between 15 and 20 passages were used in the study.

### Expression of renal progenitor cell markers in MRPC

MRPC expressed Oct-4, Pax-2, α-SMA and vimentin but not E-cadherin as shown by the immunocytochemistry assay (Figure 1B). Furthermore, MSC from the bone marrow of C57BL/6 mice (mMSC) were isolated to identify the different phenotypes between mMSC with MRPC. Many markers of renal progenitors were expressed in MRPC but not mMSC as assessed by RT-PCR, including Oct-4, Pax-2, Wnt-4 and WT-1. However, CD-34 and Sca-1 were expressed in mMSC but not MRPC (Figure 1C). These results indicated that MRPC are kidney progenitor cells.

### Differentiation potential of MRPC

The *in vitro* differentiation capacity of MRPC was examined to investigate further the potency of MRPC. When induced by osteogenic differentiation medium, MRPC stained positive with Alizarin Red, indicating that they underwent osteogenic differentiation *in vitro* (Figure 1D). MRPC treated with adipogenic differentiation medium showed the presence of adipocyte morphology with positive staining for Oil-Red O (Figure 1D), which indicated their ability for adipocyte differentiation. Taken together, multi-differentiation function *in vitro* showed that MRPC were pluripotent.

### Therapeutic effect of MRPC alone, MRPC/EPO or MRPC/suramin in I/R AKI mice

**Figure 2:**
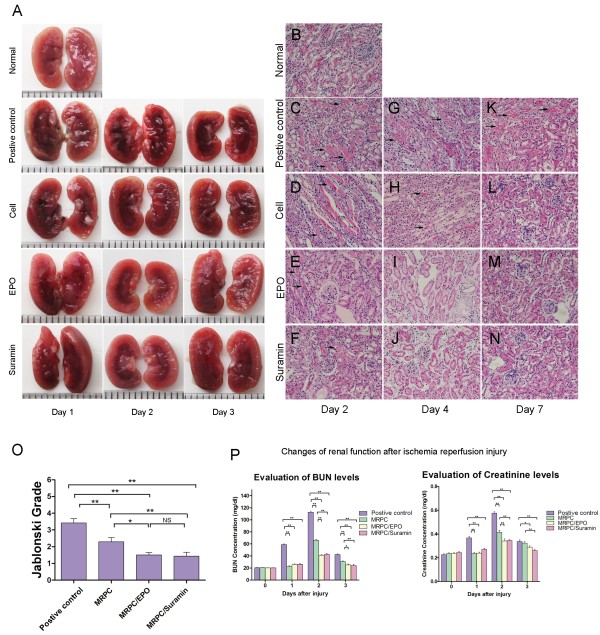
**Renal function and structure alteration after MRPC, MRPC/EPO, and MRPC/suramin injection.** (**A**) Gross morphologies of hemisected kidneys in normal or PBS- (postive control), MRPC-, MRPC/EPO-, or MRPC/suramin- treated C57BL/6 mice at one, two, and three days after ischemia-reperfusion injury. (**B**-**N**) Representative photomicrographs of **H** &**E** stained kidney sections before induction (**B**), and after two days (**C-F**), four days (**G-J**), and seven days (**K-N**) of acute renal failure in mice treated with PBS (postive control), MRPC, MRPC/EPO, or MRPC/suramin. Acute tubular necrosis induced by I/R injury involving mainly proximal tubules is seen in light micrographs. Luminal debris, loss of brush borders are observed in all groups (black arrow) (Magnification 200×). (**O**) Jablonski grading scale of histological appearance of acute tubular necrosis from mice subjected to renal ischemia treated with PBS (PC), MRPC, MRPC/EPO or with MRPC/suramin. (**P**) Serial BUN and Cr levels of acute ischemic injured mice (n = 6 in each group) in postive control, MRPC, MRPC/EPO and MRPC/suramin groups (*, *P* <0.05; **, *P* <0.01). BUN, blood urea nitrogen; Cr, serum creatinine; EPO, erythropoietin; I/R, ischemia/reperfusion; MRPC, mouse renal progenitor cells.

To investigate whether MRPC, MRPC/EPO or MRPC/suramin have beneficial effects on regeneration after AKI, renal histology and function were studied in I/R AKI C57BL/6 mice that had received tail-vein injections of MRPC, MRPC/EPO, MRPC/suramin or PBS immediately after the reperfusion. MRPC-, MRPC/EPO- and MRPC/suramin-treated mice (treatment groups) showed a reduction in the infarct zone of the injured kidney in comparison with the PBS- treated mice (positive control) (Figure 2A). Moreover, a better preservation of renal structure was shown in MRPC-, MRPC/EPO- and MRPC/suramin-treated mice (Figure 2B-N). Kidneys of the positive controls exhibited severe capillary congestion and necrosis of the tubular epithelium (Figure 2C) at day 2 and marked tubular edema and obstruction with cellular debris (Figure 2G) at day 4; and some regenerating renal tubular cells with vacuoles still appeared in the tubular injury at day 7 (Figure 2K). However, decreased histological features of necrotic injury after ischemia were sharply revealed in the kidneys of the treatment groups (Figure 2H-J). More regenerating renal tubular cells with brush border repaired tubular injury was followed by the disappearance of most necrotic tubules at day 7 (Figure 2L-N), especially in MRPC/EPO- and MRPC/suramin-treated mice. Quantitative analysis of renal tubular necrosis using the grading scores of Jablonski *et al*. [23] is shown in Figure 2O. Severe acute tubular necrosis in the kidneys of positive controls, compared to the treatment groups (especially MRPC/EPO- and MRPC/suramin-treated mice) was shown by histological grading at two days after renal ischemia (grading scores, MRPC versus positive control, MRPC/EPO versus positive control, MRPC/suramin versus positive control, *P* <0.01; MRPC/EPO versus MRPC, *P* <0.05; MRPC/suramin versus MRPC, *P* <0.01; MRPC/EPO versus MRPC/suramin, *P* >0.05).

Besides a better preservation of renal structure, improvement of renal function was observed in MRPC-, especially MRPC/EPO- and MRPC/suramin-treated mice. Serum Cr and BUN levels were measured in the treatment groups and positive controls at day 0, 1, 2 and 3. Cr and BUN reached their peak levels at day 2 of renal I/R injury in all groups. However, significantly lower levels of Cr were detected in treatment groups, especially MRPC/EPO- and MRPC/suramin-treated mice, compared to that of the positive control at day 1, 2 and 3 (Figure 2P). Taken together, MRPC alone, MRPC/EPO and MRPC/suramin were more effective in improving kidney structure and function of I/R AKI mice; MRPC/EPO and MRPC/suramin had more therapeutic effects than MRPC alone.

### Localization and roles of MRPC, MRPC/EPO and MRPC/suramin in mice with AKI

**Figure 3:**
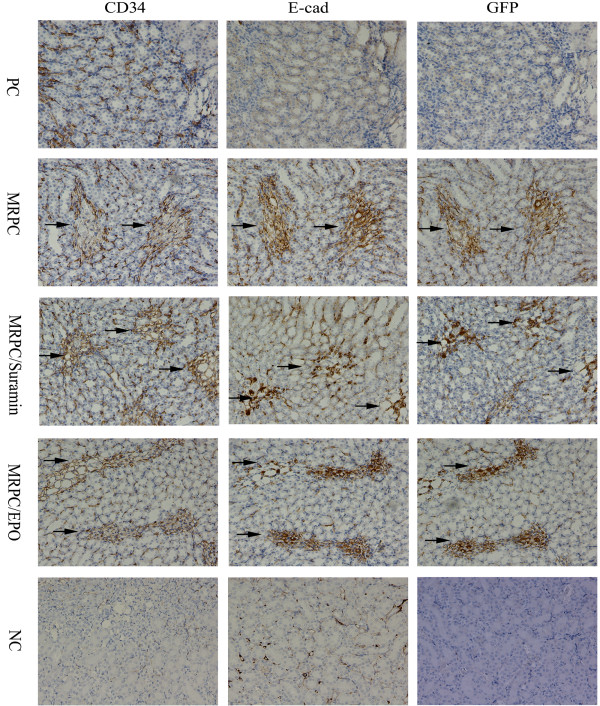
**Expression of GFP, CD34, and E-cadherin in consecutive sections of the kidney at day 2 after I/R injury.** GFP, E-cadherin and CD34 positive cells appeared in the interstitium of the kidney in the MRPC, MRPC/EPO and MRPC/suramin groups. (Magnification 200×). EPO, erythropoietin; I/R, ischemia/reperfusion; MRPC, mouse renal progenital cells.

**Figure 4:**
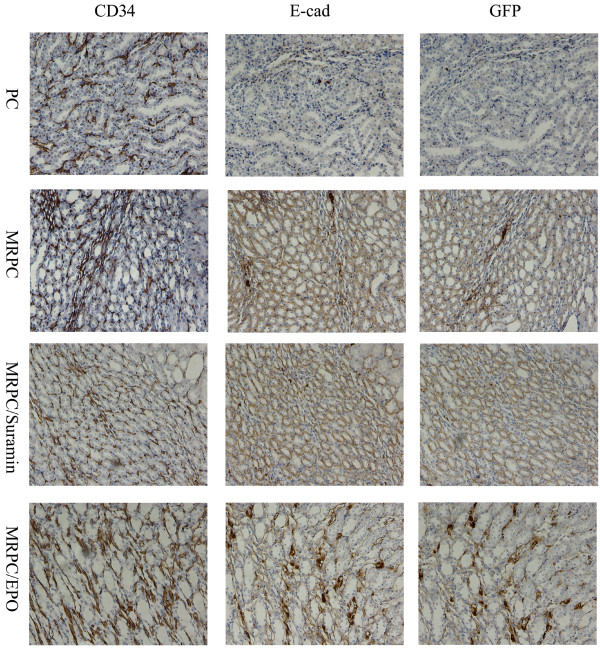
**Expression of GFP, CD34, and E-cadherin in consecutive sections of the kidney at day 4 after I/R injury.** The positive area in MRPC, MRPC/EPO and MRPC/suramin group is widely distributed. (Magnification 200×). EPO, erythropoietin; I/R, ischemia/reperfusion; MRPC, mouse renal progenital cells.

**Figure 5:**
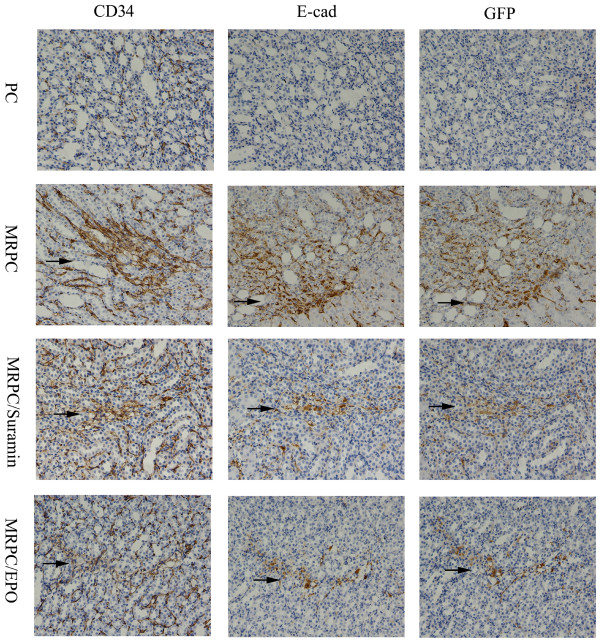
**Expression of GFP, CD34, and E-cadherin in consecutive sections of the kidney at day 7 after I/R injury.** The positive area in MRPC/EPO, MRPC/suramin group MRPC/EPO decreased, while the positive area in MRPC group was still widely distributed. (Magnification 200×). EPO, erythropoietin; I/R, ischemia/reperfusion; MRPC, mouse renal progenital cells.

It is reported that mouse kidney progenitor cells (MKPC) accelerate renal regeneration and prolong survival after ischemic injury by differentiation mechanisms in which some MKPC formed vessels with red blood cells inside (CD34^+^ cells) and some incorporated into renal tubules (E-cadherin^+^ cells) [15]. To further study the localization and roles of MRPC, MRPC/EPO and MRPC/suramin in the treatment of AKI, immunochemistry staining was performed to trace MRPC by staining GFP and analyzing the roles of MRPC, MRPC/EPO and MRPC/suramin after injection in I/R AKI C57BL/6 mice at day 2, 4 and 7 after ischemic injury (Figures 3, 4 and 5). GFP^+^ cells can become lodged in the interstitium of the kidney on day 2, 4 and 7. As shown in Figures 3, 4 and 5, CD34^+^ and E-cadherin^+^ cells were formed when MRPC, MRPC/EPO or MRPC/suramin were injected after ischemic injury. There were abundant E-cadherin and CD34 positive cells formed in the interstitium of kidney at day 2 (Figure 3). Wider distribution of E-cadherin and CD34 positive cells was shown in MRPC/EPO- and MRPC/suramin- than MRPC- treated groups at day 4 (Figure 4). The positive area decreased in the MRPC/EPO and MRPC/suramin groups, while it still remained wide in the MRPC group at day 7 (Figure 5). These results revealed that MRPC/EPO and MRPC/suramin promoted renal function recovery very early (day 2) after injection with their fast incorporation into renal tubules and capillaries; however, MRPC alone played a sustaining renal repair role in I/R AKI C57BL/6 mice.

## Discussion

Ischemic reperfusion injury is one of the main causes of AKI and more attention has been focused on stem cell therapy for ameliorating this injury. There has been mounting evidence for the existence of stem cells in the adult kidney, including the glomerulus [22], interstitium [21,24-26], tubules [8,27], and papilla [28]. In this paper we demonstrated protective roles of MRPC, MRPC/EPO and MRPC/suramin after injection in I/R AKI C57BL/6 mice. MRPC, spindle-shaped with a large nucleus, were purified from the kidneys of adult C57BL/6-gfp mice (see Additional file 1 and Additional file 2: Figure S2). They exhibited features of renal progenitor cells with expression of renal progenitor markers Oct-4 and Pax-2, Wnt-4 and WT-1, which are expressed in the renal progenitors of metanephric mesenchyme during embryonic development [29]. MRPC possessed the mesenchymal markers vimentin and α-SMA but not the epithelial marker E-cadherin. Furthermore, there was no expression of hematogenous or endothelial progenitor cell markers in MRPC, such as CD45 or CD34, which negated the possibility that MRPC originated from extrarenal tissues. In addition, MRPC were multipotent for their differentiation into osteoblast and adipocyte lineages *in vitro* and *in vivo* (see Additional file 1 and Additional file 3: Figure S3). Moreover, we studied the roles of MRPC alone and in combination with EPO or suramin in the I/R AKI mice model. In agreement with previous studies that showed that MKPC accelerate renal regeneration and prolong survival after ischemic injury [15,21], these findings identify a suitable cell population, MRPC, for possible use in future studies of cell therapy for AKI. Here, we found that the effect of MRPC/EPO or MRPC/suramin was considerably stronger than MRPC alone very early (day 2) after injection. However, MRPC alone played a sustaining renal regeneration role in I/R AKI C57BL/6 mice. The reasons for this difference still remain to be clarified. A possible explanation is MRPC/EPO or MRPC/suramin formed more CD34^+^ and E-cadherin^+^ cells with fast incorporation into renal tubules and capillaries than MRPC alone, consistent with differentiation mechanisms that some MKPC formed vessels with red blood cells inside (CD34^+^ cells) and some incorporated into renal tubules (E-cadherin^+^ cells) [15].

However, MRPC alone played a sustaining renal regeneration role in I/R AKI C57BL/6 mice. The reasons for this still remain to be clarified. It is interesting that whether MRPC homed to the injured region. Our results showed that, seven days after ischemic injury and MRPC injection, GFP fluorescence was detected in some tubules of the kidney by immunofluorescence. One possible explanation may be based on the damaged vascular system in I/R AKI C57BL/6 mice. Acute ischemic injury of the kidney induced hypoxia in the injured region and, therefore, upregulated the expression of SDF-1 which attracted CXCR4^+^ cells (MRPC) to mobilize to the injured region [30]. As the renal protection effect of MRPC was fast and immediate, there may be many mechanisms involved in the recovery process. Reduction of the inflammatory response was considered as a possible mechanism in the treatment of AKI. It was found that MRPC reduced the post-ischemic inflammatory response and obviously decreased macrophage infiltration, especially when combined with EPO or suramin (see Additional file 1 and Additional file 4: Figure S4).

How MRPC combine with EPO or suramin in the treatment of AKI is still not fully understood. As we know, EPO, a glycoprotein hormone, can stimulate the formation and differentiation of erythroid precursor cells in the bone marrow. However, further studies have been done on the undiscovered roles of EPO on other cell types that express EPO receptors [31-33]. Recent studies have shown that there are EPO receptors on the surfaces of tubular epithelial cells [31,34]. Furthermore, EPO plays an important role in these cells to protect kidneys against acute injury in animal studies [31-33,35]. Mechanisms involved in this protection appear to be associated with anti-apoptotic, anti-oxidative and anti-inflammatory properties as well as with the proangiogenic potential of EPO [31]. It was reported that rhEPO treatment significantly attenuated the upregulation of transforming growth factor 1 (TGF-1) and α-SMA and the downregulation of E-cadherin in the obstructed kidney in a mouse model [36]. Further, EPO treatment can increase the expression of CD34 [37] after adriamycin-induced kidney injury. Moreover, E-cadherin is highly positively regulated by EPO in a PI3K-dependent manner in CD34^+^ progenitor cells [38]. These findings may explain the greater improvement in renal histology and function in the mice treated with MRPC/EPO than in those treated with MRPC alone very early after injection. Suramin, a common drug in the treatment of trypanosomiasis, has recently been found to be useful in accelerating kidney recovery after AKI although the exact mechanism is still incompletely known. Recently, it was reported that the death of renal epithelial cells could directly cause necrosis of renal fibroblasts by releasing ATP immediately into the interstitium of the kidney as a death factor and the P2X_7_ receptor as a crucial mediator [39]. Since peritubular fibroblasts in the kidney are the major EPO-producing cells, inhibition of P2X_7_ may promote renal structural and functional recovery after AKI. Since suramin is a general P2 inhibitor, it can inhibit the P2X_7_ receptor to prevent the death of renal fibroblasts and then raise the EPO level during the AKI process. Thus, suramin may protect against kidney injury by increasing EPO production. There is a close intrinsic correlation between EPO and suramin. However, it is still unclear how MRPC combine with EPO or suramin in the treatment of AKI and advanced research work needs to be done.

Recently, some studies have proven that the therapeutic efficiency of MSC in AKI and many other diseases may be improved by combination with a molecular treatment. La Manna *et al*. [40] showed that hyaluronan monoesters with butyric acid (HB) act as a preconditioning agent increasing angiogenesis and vascular regeneration efficiency of FMhMSCs. Mias *et al*. [41] found that pretreatment with melatonin could increase the survival, paracrine activity and efficiency of MSCs. Similarly, the protective effects of EPO compounds and MSC combinations are supported by a study which evaluated the effect of this combination on a rat model of ischemia [42]. Although these data are from MSC, it is still reasonable to speculate that the efficiency of MRPC may also be enhanced by combination with molecular treatment. Our data show that MRPC treatment was an efficient approach for recovery from injury. There was no teratoma formed in the kidney six weeks after MRPC injection (see Additional file 1), and there are currently no reports about tumor genesis originating from MRPC. Moreover, our data show that combined MRPC/EPO and MRPC/suramin treatment was a more efficient approach for recovery from injury than MRPC alone very early (day 2) after injection and that MRPC alone played a sustaining renal repair role in I/R AKI C57BL/6 mice. Even though this potentiated effect might be related to the addition of independent beneficial effects of the treatment agents, combination of stem cell-based therapy with pharmacy therapy might offer a novel therapeutic approach for the treatment of I/R-induced AKI in humans.

## Conclusions

Taken together, our data suggest that MRPC, generated from the kidney of C57BL/6-gfp mice, might provide a new approach for the treatment of AKI in an *in vivo* model of acute kidney injury. Our results also indicate that MRPC/EPO or MRPC/suramin provided more beneficial effects very early (day 2) after injection, while MRPC alone played a sustaining role in renal regeneration in the treatment of I/R AKI. These findings suggest that it is feasible to rescue renal damage by the injection of MRPC alone, MRPC/EPO or MRPC/suramin in mice.

## Abbreviations

BUN: Blood urea nitrogen; Cr: Serum creatinine; DAPI: 4,6-diamino-2-phenyl indole; (D)MEM/F12: (Dulbecco’s) modified Eagle’s medium/F12; EPO: Erythropoietin; FBS: Fetal bovine serum; GFP: Green fluorescent protein; H & E: Hematoxylin and eosin; IgG: Immunoglobulin G; I/R AKI: Ischemia/reperfusion acute kidney injury; MKPC: Mouse kidney progenitor cells; MRPC: Mouse renal progenitor cells; MSC: Mesenchymal stem cells; PBS: Phosphate-buffered saline; RT-PCR: Reverse transcription polymerase chain reaction; SMA: Smooth muscle actin.

## Competing interests

The authors declare that they have no competing interests.

## Authors’ contributions

XH participated in manuscript writing, data analysis and interpretation, collection and assembly of data. LZ participated in collection and assembly of data, data analysis and interpretation. GL and JG participated in collection and assembly of data. YZ participated in data analysis and interpretation. SZ participated in collection and assembly of data. MY and YL participated in the provision of study material. FK participated in administrative support. ZX participated in manuscript writing. SZ participated in conception and design, financial support and final approval of manuscript. All authors read and approved the final manuscript for publication.

## Supplementary Material

Additional file 1 **Supplementary data.** Supplementary material and method; Supplementary results; Supplementary figure legend. Click here for file

Additional file 2: Figure S2 Fluorescence intensity of MRPC. Fluorescence intensity of new isolated MRPC and MRPC cultured for 4 weeks detected by FACS. Fluorescence intensity of cells prepared from GFP transgenic mouse was much stronger than cells from C57BL/6 mice. MRPC isolated from normal c57bl/6 mice as control. Click here for file

Additional file 3: Figure S3 *In vivo* differentiation potency of MRPC. *In vivo* differentiation of MRPC seven days and six weeks after MRPC injection. MRPC incorporated into Henle’s loop by expressing Tamm-Horsfall glycoprotein in the medulla. Six weeks after the injection, more GFP positive cells could be detected than day seven. Immunofluorescence staining of Henle’s loop marker Tamm-Horsfall glycoprotein (red), fluorescent MRPC (green), nuclear are stained with DAPI (blue) (Magnification 400×). Click here for file

Additional file 4: Figure S4 Inflammatory cell infiltration. Immunofluorescence of macrophage infiltration stained with anti-F4/80 antibody (red) one, two and three days after ischemia-reperfusion injury in the kidney treated with PBS (postive control), with MRPC, with MRPC/EPO, or with MRPC/suramin. Nuclears are stained with DAPI (blue) (Magnification 400×). Click here for file
