# Supplementary material for: Improving outcomes of acute kidney injury using mouse renal progenitor cells alone or in combination with erythropoietin or suramin
Source: Stem Cell Res Ther. 2013 Jun 18;4(3):74. doi: 10.1186/scrt225 (PMC3706945; doi:10.1186/scrt225)
Supplement: Additional file 1 — Supplementary data. Supplementary material and method; Supplementary results; Supplementary figure legend. [file scrt225-S1.docx]

**Supplementary material and method.**

**Cell Isolation and Culture**

The purification of MRPC was done by reduplicative cell passage [8] and detecting the level of green ﬂuorescence intensity by FACS. After 4 weeks, most cell types died out and the cultures became monomorphic with spindle-shaped cells. Moreover, the green ﬂuorescence intensity increased after cell passage.

**Characterization of MRPC**

***Differentiation in vivo.*** The in vivo differentiation of MRPC was studied in I/R AKI mouse model. 10^5^ MRPC in 50 µl PBS were slowly injected via tail vein injection. 7 days and 6 weeks later, the kidneys were harvested to examine the in vivo differentiation of the injected MRPC. The differentiation of MRPC was detected by expression of Henle’s loop marker Tamm-Horsfall glycoprotein (THG) (Santa Cruz, sc-19554, 1:50) using immunofluorescence staining.

**Effect of MRPC on Renal Protection after Acute Ischemic injury**

***Study design.*** Teratoma formation was detected by gross examination and HE staining 6 weeks after MRPC injection sub-renal capsule.

***Surgical procedure.*** 5×10^5^ MRPC were injected via tail vein for this dosage is safe and effective in all process.

***Immunofluorescence.*** Inflammatory cells infiltration was detected by immunofluorescence in PBS-, MRPC-, MRPC/EPO- or MRPC/Suramin- treated mice on day 1, 2, 3. Briefly, after harvest, kidneys were fixed in 4% PFA for 4 hours, and dehydrated successively in graded sucrose (10%, 15% and 20%) for 2 hours respectively. These kidneys were embedded with OCT compound (Sakura Finetek USA, Torrance, CA). Cryosections 4 µm thick were collected onto slides and stored at –80°C before immunolabeling. Immunofluorescence assay was done as before. After blocking with 4% normal goat serum in PBS, the slides were stained with primary antibodies overnight at 4℃, secondary antibody for 60 minutes at 37 ℃. Rabbit monoclonal anti-F4/80 primary antibody (Abcam, ab6640, 1:20) was used.

**Supplementary results**

**Isolation and culture of fluorescent MRPC**

The FACS results showed that fluorescence intensity of cells prepared from GFP transgenic mouse was much stronger than cells from C57BL/6 mice. Moreover, the green ﬂuorescence intensity increased after cell passage (see Additional file 2: Figure S2).

**Differentiation potential of MRPC**

In vivo differentiation capacity of MRPC was performed to examine whether MRPC have the potential to differentiate into functional renal cells. 7 days after the initial injection, scattered MRPC incorporated into Henle’s loop by expressing Tamm-Horsfall glycoprotein in the medulla (see Additional file 3: Figure S3). And 6 weeks after the injection, more GFP positive cells could be detected than day 7, the tubule incorporated with GFP positive MRPC were stained with Henle’s loop marker Tamm-Horsfall glycoprotein (THG) (see Additional file 3: Figure S3).

**Therapeutic effect of MRPC alone, MRPC/EPO or MRPC/Suramin in I/R AKI mice**

MRPC were found that they reduced post-ischemic inflammatory response, MRPC decreased macrophage infiltration obviously, especially when combined with EPO or suramin (see Additional file 4: Figure S4).

Furthermore, whether allograft infusion of C57BL/6-gfp MRPC into C57BL/6 mice retards the possible clinical application of this therapy was a great concern. Teratoma formation was detected by gross examination and HE staining 6 weeks after MRPC injection sub-renal capsule. There was no teratoma formed 6 weeks after injection.

**Supplementary figure legend**

**Supplementary Figure S2 Fluorescence intensity of MRPC.** Fluorescence intensity of new isolated MRPC and MRPC cultured for 4 weeks detected by FACS. fluorescence intensity of cells prepared from GFP transgenic mouse was much stronger than cells from C57BL/6 mice. MRPC isolated from normal c57bl/6 mice as control.

**Supplementary Figure S3 In vivo differentiation potency of MRPC.** In vivo differentiation of MRPC 7 days and 6 weeks after MRPC injection. MRPC incorporated into Henle’s loop by expressing Tamm-Horsfall glycoprotein in the medulla. 6 weeks after the injection, more GFP positive cells could be detected than day 7. Immunofluorescence staining of Henle’s loop marker Tamm-Horsfall glycoprotein (red), fluorescent MRPC (green), nuclear are stained with DAPI (blue) (Magnification 400×).

**Supplementary Figure S4 Inflammatory cell infiltration.** Immunofluorescence of macrophage infiltration stained with anti-F4/80 antibody (red) 1, 2, and 3 days after ischemia-reperfusion injury in the kidney treated with PBS (postive control), with MRPC, with MRPC/EPO, or with MRPC/Suramin. Nuclears are stained with DAPI (blue) (Magnification 400×).
