# Supplementary figures and images for: Improving outcomes of acute kidney injury using mouse renal progenitor cells alone or in combination with erythropoietin or suramin
Source: Stem Cell Res Ther. 2013 Jun 18;4(3):74. doi: 10.1186/scrt225 (PMC3706945; doi:10.1186/scrt225)

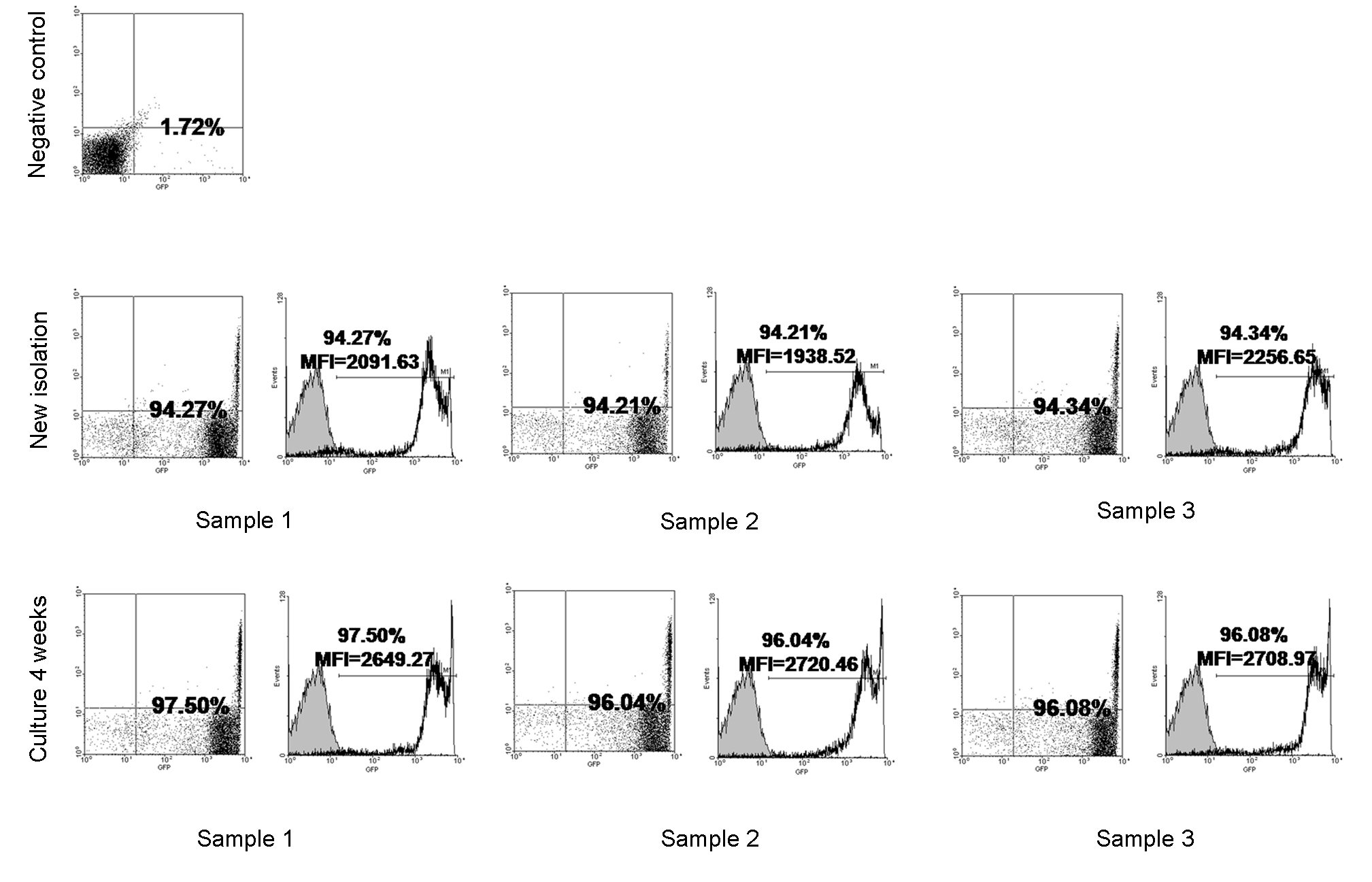

Supplement: Additional file 2: Figure S2 — Fluorescence intensity of MRPC. Fluorescence intensity of new isolated MRPC and MRPC cultured for 4 weeks detected by FACS. Fluorescence intensity of cells prepared from GFP transgenic mouse was much stronger than cells from C57BL/6 mice. MRPC isolated from normal c57bl/6 mice as control. [file scrt225-S2.jpeg]

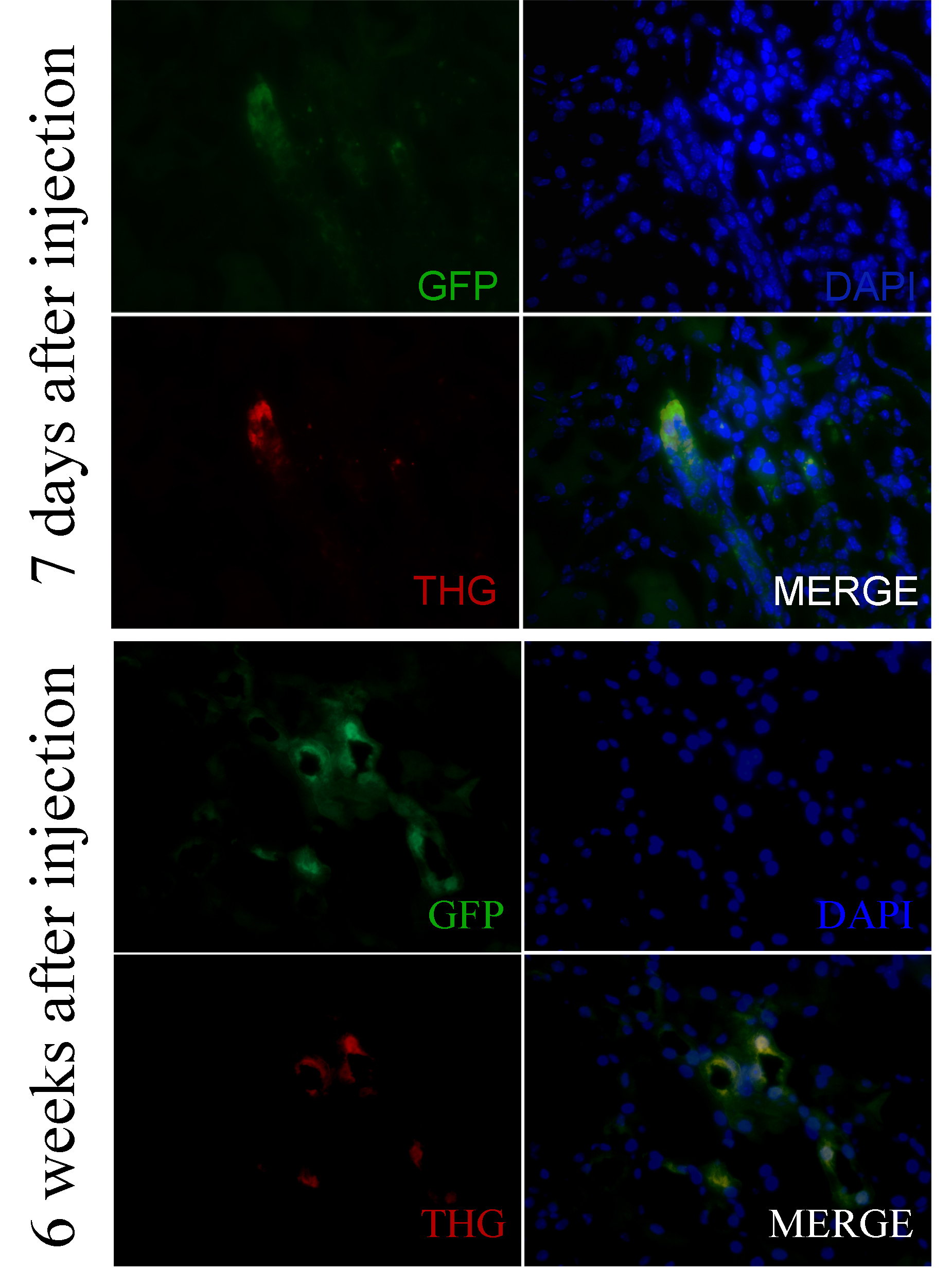

Supplement: Additional file 3: Figure S3 — In vivo differentiation potency of MRPC. In vivo differentiation of MRPC seven days and six weeks after MRPC injection. MRPC incorporated into Henle’s loop by expressing Tamm-Horsfall glycoprotein in the medulla. Six weeks after the injection, more GFP positive cells could be detected than day seven. Immunofluorescence staining of Henle’s loop marker Tamm-Horsfall glycoprotein (red), fluorescent MRPC (green), nuclear are stained with DAPI (blue) (Magnification 400×). [file scrt225-S3.jpeg]

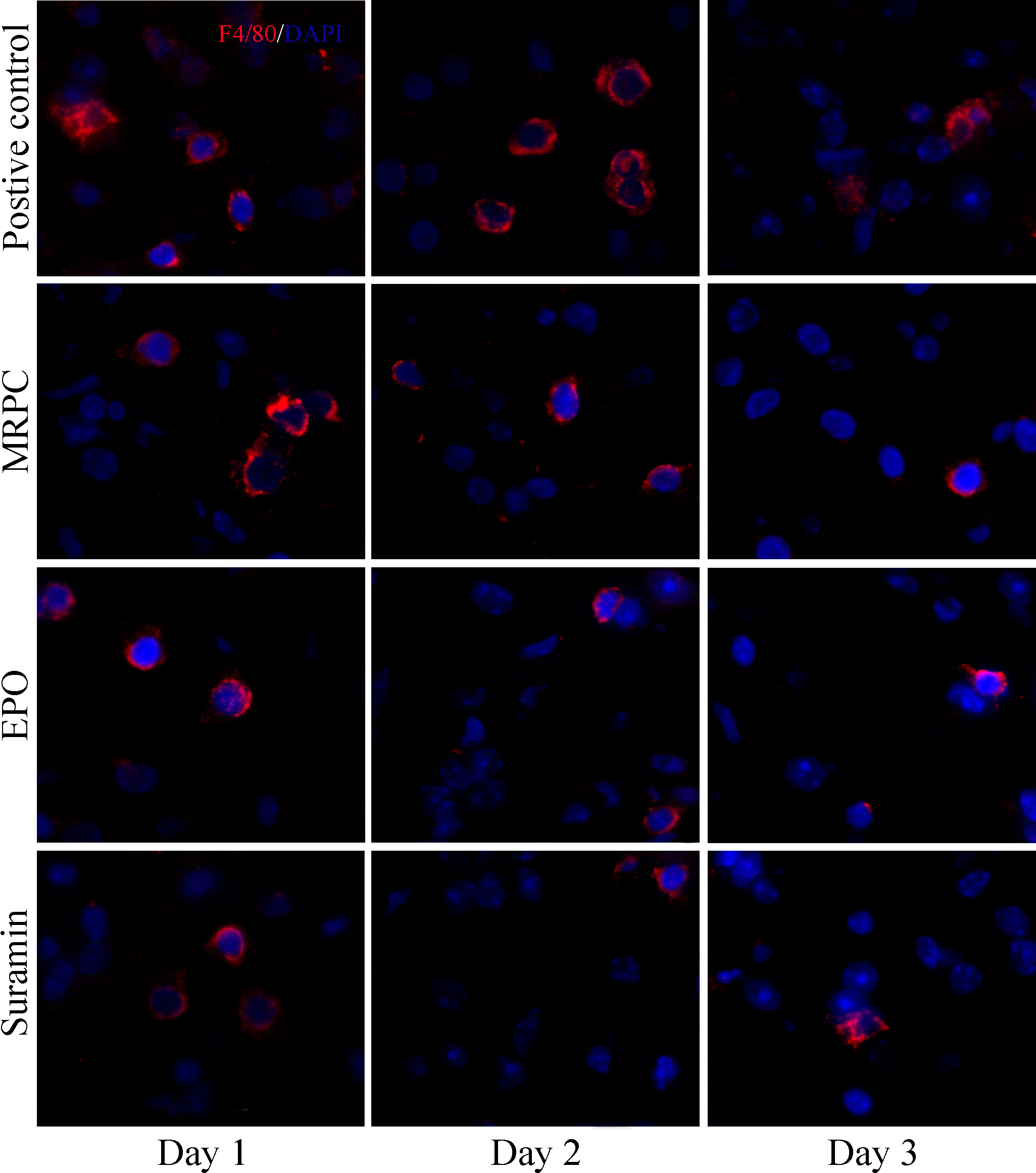

Supplement: Additional file 4: Figure S4 — Inflammatory cell infiltration. Immunofluorescence of macrophage infiltration stained with anti-F4/80 antibody (red) one, two and three days after ischemia-reperfusion injury in the kidney treated with PBS (postive control), with MRPC, with MRPC/EPO, or with MRPC/suramin. Nuclears are stained with DAPI (blue) (Magnification 400×). [file scrt225-S4.jpeg]
